# A Combined Deep Learning GRU-Autoencoder for the Early Detection of Respiratory Disease in Pigs Using Multiple Environmental Sensors

**DOI:** 10.3390/s18082521

**Published:** 2018-08-02

**Authors:** Jake Cowton, Ilias Kyriazakis, Thomas Plötz, Jaume Bacardit

**Affiliations:** 1 Interdisciplinary Computing and Complex BioSystems (ICOS) Research Group, School of Computing, Newcastle University, Newcastle upon Tyne NE1 7RU, UK; jaume.bacardit@newcastle.ac.uk; 2 Agriculture, School of Natural and Environmental Sciences, Newcastle University, Newcastle upon Tyne NE1 7RU, UK; ilias.kyriazakis@newcastle.ac.uk; 3 Open Lab, Newcastle University, Newcastle upon Tyne NE1 7RU, UK; thomas.ploetz@gatech.edu

**Keywords:** anomaly detection, deep learning, sensors, GRU, pig, health, disease

## Abstract

We designed and evaluated an assumption-free, deep learning-based methodology for animal health monitoring, specifically for the early detection of respiratory disease in growing pigs based on environmental sensor data. Two recurrent neural networks (RNNs), each comprising gated recurrent units (GRUs), were used to create an autoencoder (GRU-AE) into which environmental data, collected from a variety of sensors, was processed to detect anomalies. An autoencoder is a type of network trained to reconstruct the patterns it is fed as input. By training the GRU-AE using environmental data that did not lead to an occurrence of respiratory disease, data that did not fit the pattern of “healthy environmental data” had a greater reconstruction error. All reconstruction errors were labelled as either normal or anomalous using threshold-based anomaly detection optimised with particle swarm optimisation (PSO), from which alerts are raised. The results from the GRU-AE method outperformed state-of-the-art techniques, raising alerts when such predictions deviated from the actual observations. The results show that a change in the environment can result in occurrences of pigs showing symptoms of respiratory disease within 1–7 days, meaning that there is a period of time during which their keepers can act to mitigate the negative effect of respiratory diseases, such as porcine reproductive and respiratory syndrome (PRRS), a common and destructive disease endemic in pigs.

## 1. Introduction

The forecasting of an oncoming health challenge in animals is fundamental to maintaining a high level of health and animal welfare. The earlier a disease is predicted, the sooner it can be dealt with, thus lowering the overall impact of the disease on both the animal and the farm, and increasing the likelihood of treatment success [1]. Respiratory diseases, such as porcine reproductive and respiratory syndrome (PRRS), pneumonia, and pleurisy, are some of the most common types of disease found in commercial pig populations [2]. Therefore, it is highly valuable to be able to predict occurrences of respiratory disease, which we define as the point in time where respiratory disease prevalence increases from 0 to greater than 0.

There are many factors that influence the contraction of respiratory diseases, in particular, the environment which the pigs inhabit. A number of the respiratory disease risk factors discussed by Stark et al. [2]—specifically, air quality, temperature, and humidity—are now able to be monitored continuously in real time and at a high resolution using inexpensive electronic sensors. Such data can be used to further understand the relationship between environmental conditions and respiratory disease.

In most situations, recording data continuously means storing data that predominantly describes normal circumstances, as anomalous events are less frequent. The large amounts of normal data generated allow us to train a solid model of normality. Hence, it is better to model normality and then quantify any deviations from it.

The objective of this paper is to describe and evaluate a methodology for the early warning of anomalies pertaining to respiratory disease in growing pigs, based on environmental factors. A recurrent autoencoder, built using deep learning (gated recurrent units (GRU)), learns how to reconstruct raw sensor data which does not lead to occurrences of respiratory disease within a batch of pigs. This GRU-autoencoder (GRU-AE) can then be used to “score” a window of sensor data according to how close it is to normality. A threshold-based anomaly detector that was optimised using particle swarm optimisation (PSO) used this score to determine whether to raise an alert for scores which are too great and therefore represent environmental conditions that do not fit in line with the data it was trained to represent.

The benefits of using such a method are that there is no need for hand-crafted features, as is typically required in classical statistical approaches, meaning that raw sensor data can be used with minimal preprocessing. Also, no arbitrary thresholds are set, as the detection of anomalous events is wholly data-driven. In addition, there is no requirement for an in-depth understanding of the relationship between environmental conditions and pig health, as this is handled implicitly within the network. This work is structured as an anomaly detection solution, rather than, for example, predicting increases in respiratory disease prevalence; therefore, the method is able to perform on data consisting of low disease prevalence without affecting performance. However, the more cases of disease there are, and the higher the prevalence at these times, the easier it is to fine-tune and validate the method.

Malhotra et al. [3] demonstrated the potential of a long short-term memory (LSTM)-based encoder-decoder structure for multi-sensor anomaly detection, showing the methodology has good potential for using data which is not easily predictable [4]. This implementation used LSTM cells, rather than a standard artificial neural network (ANN) structure, in order to handle the temporal dimension, and used reconstruction error was used to determine anomalies. However, the results of their implementation on various datasets suffered distinctly either in precision or recall (defined in Section 4.1.1). This paper revisits the design choices made in previous work, such as that by Malhotra et al., specifically by:

- exchanging long short-term memory cells for GRU cells
- introducing particle swarm optimisation to optimise an anomaly detector that determines whether the loss from the GRU-AE is anomalous
- changing how the overall system is evaluated to demonstrate a more balanced anomaly detection system

The remainder of the paper is structured as follows. Firstly, Section 2 gives context for the approach taken. Section 3 explains the composition of the data and the preprocessing that was applied, followed by Section 4, which details how each of the models were constructed. Section 5 outlines the performance of the systems described, followed by the discussion in Section 6 and the conclusions drawn in Section 7.

## 2. Background

In recent years, agricultural research has begun to shift away from relying only on classical statistical approaches and started to regularly incorporate machine learning (ML) methodologies, such as Support Vector Machines (SVM) and Random Forest Classifiers, into the approach to data analysis [5,6,7,8,9,10].

Recent work has seen SVMs being used, for example, to detect anomalies in the production curve of eggs produced by commercial hens, covering a range of prediction windows for the purpose of an early warning system [5]. Other research has applied ensemble learning methods (combined models) for the prediction of avian influenza prevalence using meteorological data, showing improved performance over support vector regression [6].

Classification methods have been used to separate normality from abnormality through the implementation of one-class classifiers (OCCs). SVM-based methods are very common in this approach, though there are many potential methods which can be applied. Models of this type usually have high sensitivity but a comparatively poor specificity [8], which is to be expected given the natural imbalance in the datasets that these methodologies are commonly used for. However, these metrics are not always suitable, particularly if the true negatives drastically outweigh the number of false positives. Cabral et al. compared a number of common OCCs with the proposal of features boundary detection for one-class classification (FBDOCC) of heart disease [8].

There are also approaches to anomaly detection outside of classification, such as clustering [11] and correlation analysis [12]. Clustering uses distance metrics to determine if a new point’s distance from a cluster centroid exceeds an acceptable value: if this is the case, it is considered anomalous. However, methods falling into this category are typically unable to strike a reasonable balance between quality of analysis and speed due to the innate characteristics of time-series data [13]. Correlation analysis uses similarity metrics to determine if new data is similar to known normal data.

Anomaly detection can be used in health applications to detect outbreaks of disease or other deviations from normality. A common methodology used for this task are Bayesian networks, which have been shown to outperform classical statistical methods, such as control charts, moving average models, and ANOVA regression [14]. In one simulation of an anthrax outbreak [15], the Bayesian network was able to correctly trigger an alarm within 2 days, in comparison to 9 days for the moving average model and 11–12 days for the others. Anomaly detection is very well suited to healthcare, as often there are not necessarily certain conditions that constitute a problem, but rather deviations from certain conditions. For example, when detecting arrhythmia through electrocardiography (ECG), it is not possible to have knowledge of all of the different types, as new types can occur in the future [16]. It is therefore necessary to understand what normality looks like, rather than what problems look like.

In health applications where sensors are used, there are two main challenges. Firstly, there is a need to focus not only on a high detection rate, but also a low false positive rate [17], as, if this is high, alerts from this system will not be trusted. Secondly, though not in all cases, anomalies ought to be detected in near real time [18] in order to provide a quick response so that interventions can be carried out. This creates a substantial limitation on which methods can be used, as some are very resource intensive.

Often, these approaches require a transformation be carried out on the data in order to generate a useful feature representation [19,20,21]. However, deep learning-based methods benefit by not requiring this step. Raw data can be passed to a deep network without any hand-crafting of features, as this is handled intrinsically by the network [16]. However, this requires a substantial amount of data to function effectively [22]. This is particularly valuable for high-dimensional data, as, provided the network is deep enough to model the data, all data can be utilised.

A relatively small amount of work in agriculture has made use of emerging technologies, such as deep learning, which mostly focuses on image analysis and remote sensing [23]. This paper demonstrates how deep learning methods, used in conjunction with evolutionary algorithms, can be used for time-series analysis for the purpose of anomaly detection.

**Figure 1:**
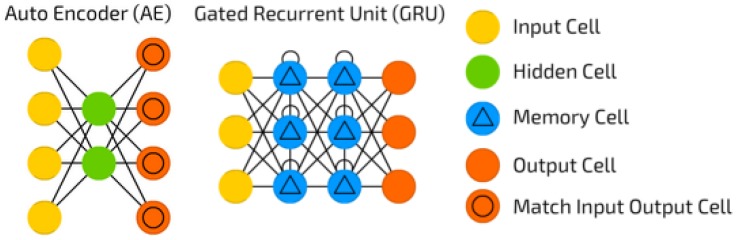
A visualisation of a classic autoencoder (**left**) and recurrent neural network consisting of gated recurrent units (GRUs) (**right**) [24]. These two methodologies are what we combined to create an autoencoder (AE) capable of utilising temporality.

Autoencoders are artificial neural networks (ANNs) that are trained to reconstruct input patterns (Figure 1). Given an input tensor *N*, an encoding *M* is found which is used to create $N^{\prime }$. The network is trained to minimise the loss between *N* and $N^{\prime }$. The transformation of *N* to *M* is the encoder, and the attempted reconstruction of *M* to $N^{\prime }$ constitutes the decoder. Autoencoders have shown good performance in assisting anomaly detection, often through learning the feature representation *M* and using it as the input to an OCC [25,26,27], though some papers have made use of the reconstruction error to detect anomalous data [28].

## 3. Experimental Design

### 3.1. Data Collection

Data was collected for growing pigs from a variety of farms across Europe as part of an EU-funded multidisciplinary project. Each farm was represented by a number of batches of pigs (3–16 batches), where a batch consists of varying population sizes (40–1294 pigs), and the batches were monitored for varying lengths of time (14–145 days). The starting weight of the pigs was variable, ranging from 17.5 to 27.6 kg. The number of pigs per batch affected by respiratory disease was recorded on a daily basis. Respiratory disease prevalence was divided into four levels of severity, as defined by Zoetis’s Individual Pig Care [29]. A pig could be either scored (identified as diseased but no treatment given) or treated for each level of severity. Health data regarding the number of pigs showing symptoms of disease was collected by a trained person. They manually counted the number of pigs in each batch that showed symptoms of certain diseases and classified it by the level of severity into which the symptoms fell. Given the nature of the system, pigs cannot be identified individually each day, so the number of pigs that are classified as showing symptoms can be new cases or pigs that were identified as such in the previous day.

The environmental data was collected using General Alert [30] sensors, which were suspended in the centre of the room in which the batch was located. The sensors recorded the environmental features relevant to pig health [31]. Briefly, the system monitored temperature (a sensor range of −50 °C to +250 °C ± 0.05%), relative humidity (a sensor sensitivity of ±2% and operating temperature range of −40 °C to +85 °C), and CO${}_{2}$ concentration (with a range from 0 ppm to 5000 ppm ± 30 ppm or ±3% of reading, operating temperature range from 0 °C to 50 °C, and humidity from 0% to 95%), which was sampled every minute.

### 3.2. Data Preprocessing

**Figure 2:**
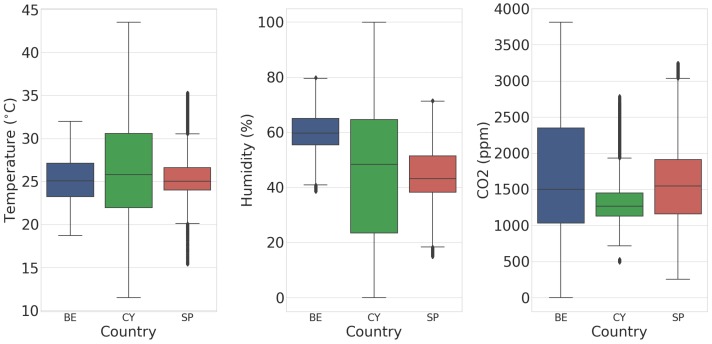
A boxplot describing the temperature, humidity, and CO${}_{2}$ sensor data for the three countries included in the data collection—Belgium (BE), Cyprus (CY), and Spain (SP)—after preprocessing.

Outliers were determined to be anything outside μ±2σ per variable per batch and were removed from the data. Missing data induced both from the removal of outliers and due to hardware issues were filled using linear interpolation. This was deemed acceptable, as the environment has low short-term variance, meaning that, on average, the assumption of a linear change between two values was reasonable. The results of this preprocessing of sensor data are shown in Figure 2. The three sensor readings—temperature, humidity, and CO2—constitute the inputs to the models outlined in Section 4. The four severity levels were summed and used to calculate the proportion of pigs showing symptoms of respiratory disease on a given day, rather than looking at one specific level of severity (Equation (1)).

(1) $$\mathrm{prevalence}=\frac{\mathrm{Number}\;\mathrm{of}\;\mathrm{pigs}\;\mathrm{showing}\;\mathrm{any}\;\mathrm{symptoms}}{\mathrm{Total}\;\mathrm{number}\;\mathrm{of}\;\mathrm{pigs}}$$

**Figure 3:**
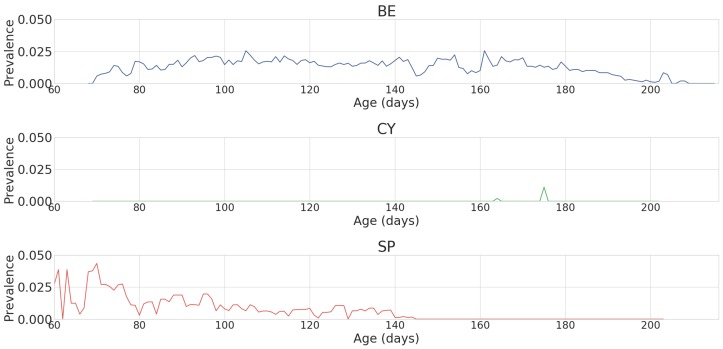
A graph showing the average prevalence of respiratory disease against age (in days) in all batches of growing pigs in Belgium (BE), Cyprus (CY), and Spain (SP) after preprocessing.

This was done because respiratory diseases rarely exceed the classification of light or mild, as in most cases they are treated before the disease develops into more severe symptoms. Therefore, there were very few cases of severe or irrecoverable respiratory disease across all farms. Even with the aggregation of data, the level of prevalence was still very low, and it decreased even further over time (Figure 3). The sensor data was merged with the daily health data by upscaling the resolution of the latter by duplicating the values for the full 24 h period. All input was normalised to between 0 and 1.

#### 3.2.1. Separation of Assumed Healthy and Assumed Unhealthy Data

As this paper investigates the effects of environment on health, it was necessary to account for the existence of a lag period between a change in environment and a change in health, as a change in environment will not immediately result in an outbreak of disease [32]. Lag periods of 14 and 21 days were both evaluated [32]; greater windows were not used, as this would greatly reduce the amount of data available for training. This lag period was referred to as the assumed unhealthy window, *u*. Using this window, the data was labelled as one of two sets: “assumed healthy” and “assumed unhealthy”. Data from any point where the proportion of pigs showing symptoms was greater than 0 to *u* days after the disease prevalence decreases to 0 was labelled “assumed unhealthy”; all other data was labelled “assumed healthy”. All data at this point was broken down into 30 min “frames” using a sliding window.

#### 3.2.2. Splitting Data into Train, Validation, and Test Sets

**Figure 4:**
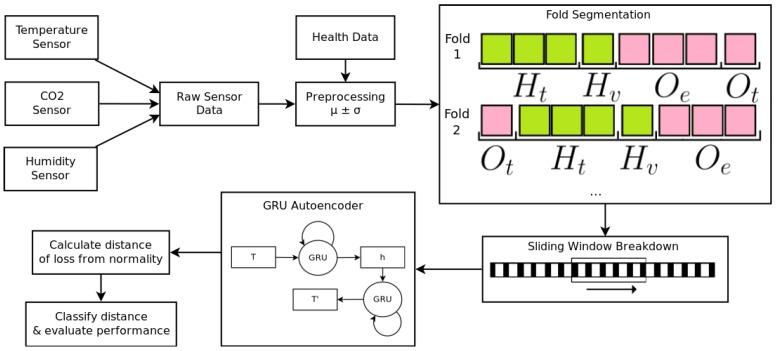
An overview of the entire processing workflow, from the raw sensor data to anomaly detection. Temperature, CO${}_{2}$, and humidity sensors were preprocessed and merged with daily health data. This was then broken down into four folds—$H_{t}$ and $H_{v}$ (used for the GRU-AE), and $O_{e}$ and $O_{t}$ (used for the anomaly detection). Each of these folds were broken down using a sliding window sized at 30 min. Each window of 30 min was processed by the GRU-AE, and a reconstruction error was produced. The distance between this reconstruction error and normality was used to detect whether the window is anomalous or not, using a threshold-based anomaly detector optimised using particle swarm optimisation.

It was necessary to split data into training and testing sets. Leave-one-batch-out cross-validation was used to achieve this (Figure 4). Our method, detailed in Section 4.1, and the GRU-regression (GRU-R) model used for comparison, detailed in Section 4.3.3, were each composed of two parts: a GRU network and a threshold-based anomaly detector. Both the GRU network and anomaly detector required two sets of data for the full workflow. $H_{t},H_{v}$ were used for the GRU network, and $O_{e}$ and $O_{t}$ were used for the anomaly detector. $H_{t}$ was used for the training of GRU networks and $H_{v}$ was used to validate it. $O_{e}$ was used to optimise an anomaly detector via PSO and $O_{t}$ was the set the anomaly detector was tested on to attain an evaluation metric. There was no overlap between any of these sets.

$O_{t}$ was a single, independent farm batch, $O_{e}$ was composed of three randomly chosen batches, $H_{v}$ contained only the assumed healthy data of another independent farm batch, and $H_{t}$ contained only the assumed healthy data from all the remaining farm batches.

Each of the four datasets could be represented as a matrix $X\in {I\;R}^{n\times 3}$, where *n* is the number of minutes of data in a farm batch, and each $X_{n}$ contains sensor data for temperature, humidity, and CO${}_{2}$. This matrix was broken down into frames using a sliding window of size *w*, which moved one timepoint (1 min) per step, creating $T\in {I\;R}^{m\times w\times 3}$, where *m* was the number of frames in the set.

#### 3.2.3. Preliminary Experiments Utilising Batch Normalisation

Batch normalisation is a deep learning technique that has shown to be very effective at training robust networks by performing a per layer normalization process on input data. Please note that the meaning of batch in this context differs from the farm batches used throughout the rest of the manuscript. In this context, batches are the subsets of the training data that are fed in blocks to the network being trained with the subsequent back-propagation-based error correction. In anomaly detection, the effect of this technique is not necessarily desirable and, in this particular application, based on preliminary experiments, was found to be detrimental to performance. This was because batch normalisation allows a model to deal with internal covariate shift by normalising each mini-batch by its mean and variance [33], meaning that the model is more capable of handling data it has never seen before. The decrease in performance when using batch normalisation showed that there is a requirement that the model be highly sensitive to slight variations in the data, and so it was not used in the final models.

## 4. Time-Series Early Warning Methods

### 4.1. GRU-Autoencoder

The implementation of an autoencoder model (Figure 4) consisted of two multi-layer GRU networks—an encoder and a decoder—that were trained simultaneously. This is instead of the traditional ANN structure (Figure 1), which does not intrinsically account for temporality in data. The encoder learned a fixed-size representation *h* of an input *T*. The quality of this representation was measured by using it to attempt a reconstruction of *T* using the decoder. The model was optimised to minimise the reconstruction error, calculated using binary cross-entropy loss, between *T* and $T^{\prime }$. *T* was reversed when calculating loss, as it creates short-term dependencies that result in an increased performance in sequence-to-sequence networks [34].

$H_{t}$ was used to train the GRU-AE using mini-batch RMSprop gradient descent [35]. This dataset contained only “assumed healthy” data, which resulted in the GRU-AE optimising towards a lower reconstruction error for this class of data and a higher reconstruction error for all other data. Hyper-parameters, hidden size, number of layers, mini-batch size, dropout, learning rate, and momentum were selected using grid search, and the parameters which resulted in the lowest reconstruction error for each fold’s $H_{v}$ were selected. The hidden size represents the amount of information the GRU-AE can store for each time series it is given. As there was no requirement for a large amount of memory for this task, the hidden size representation identified by the grid search was expected to be fairly small. The number of recurrent layers represents the capability of the GRU to create deep models and understanding of the time series. These are expensive in terms of computation and memory. The mini-batch size is how many sequences are passed forward through the network before back-propagating the loss. This is typically desired to be as large as possible to speed up training, though is entirely dependent on the other parameters and the amount of memory available. Dropout is a regularisation technique used to reduce the likelihood of over-fitting and is common in standard artificial neural networks. The learning rate and momentum are also used in standard artificial neural networks. They determine how drastically weights are changed during back-propagation. The selected hyper-parameters were then used to calculate reconstruction errors for all frames in $O_{e}$ and $O_{t}$.

Once all reconstruction errors were calculated, the threshold-based anomaly detector was used to determine whether each reconstruction error of a frame was to be considered anomalous or not. This was estimated by calculating the Mahalanobis distance (Equation (2)) between the reconstruction error of a given frame *e* and the distribution of the reconstruction errors for all frames of $H_{v}$, defined as *R*, where Σ=COV(e,R). This was then smoothed using locally weighted smoothing (LOWESS) [36]. If the smoothed distance was above some threshold τ (the optimisation of which is described in Section 4.1.1), then it was considered an anomalous frame.

(2) $$d=\sqrt{{\left(e-\bar{R}\right)}^{T}\Sigma^{-1}\left(e-\bar{R}\right)}$$

#### 4.1.1. PSO-Optimised Anomaly Detection

Once all distances were calculated for all frames, the next step was to optimise the threshold-based anomaly detector, capable of determining which frames should be considered anomalous, using PSO [37]—a metaheuristic, which uses concepts of swarm intelligence to find a set of hyper-parameters that produce a near-optimal solution. Consider the vector of Mahalanobis distances of all frames in a sequence $\vec{D}=\left\{d_{0},d_{1},d_{2},\ldots ,d_{n}\right\}$, such that each $d_{i}$ is the Mahalanobis distance between the reconstruction error of a frame at index *i* from *R*. An alert was defined as any $d_{i}:d_{i-1}<\tau \wedge d_{i}\geq \tau$. The validity of an alert was assessed by determining whether disease prevalence increased from 0 within an acceptable forecasting window starting at i+α and ending at α+β. If so, and if no other alert had been raised for this increase in prevalence, the alert was considered a true positive, else a false positive. Furthermore, consider the vector $\vec{F}=\left\{f_{0},f_{1},f_{2},\ldots ,f_{n}\right\}$ such that each $f_{i}$ contains the number of animals showing symptoms of respiratory disease at frame *i*. A false negative was determined to be any frame $i:f_{i-1}=0\wedge f_{i}>0$ for which there is no alert within i−α and i−β.

The parameters τ, α, and β of the detector were the parameters to be optimised using PSO. The particles were optimised for each fold independently in $O_{e}$, and the best values for each parameter were then tested on $O_{t}$ for a final performance evaluation. Each particle was randomly initialised with three dimensions representing τ, α, and β. The fitness function (Equation (3)) used by the PSO was designed to maximise both Matthews correlation coefficient MCC (Equation (4)) and α whilst, at the same time, minimising β. α and β were normalised between 0 and 1 when entered into the fitness function.

(3) F=2·MCC+α−β

(4) $$MCC=\frac{TP\times TN-FP\times FN}{\sqrt{\left(TP+FP)(FP+FN)(TN+FP)(TN+FN\right)}}$$

MCC is a measure designed to evaluate the predictions of binary classifiers, which is especially robust in problems with high class imbalance, such as our dataset. MCC scores 1 for perfect classification, −1 for perfect misclassification, and 0 for equivalent to random classification. The F-measure is commonly used in information/document retrieval, as it does not fall prey to the same problems, however, it does not include true negatives in the calculation. This is not an issue within document retrieval, as they are not important to that particular task; however, in a detection system, correctly classifying negatives (true negatives) is an integral objective of the system.

It was desirable to attain a high α, as this corresponds to how far in advance the alert of oncoming disease is raised, whereas β needs to be minimised, as this represents the period of time the alert is for. For example, given the values α=7 and β=1, we know that when an alert is raised, it is expected that occurrences of respiratory disease will appear within 7–8 days. Given a different detector for the values α=3 and β=5, we know that when an alert is raised, it is expected that occurrences of respiratory disease will appear within 3–8 days, a larger window and, therefore, more uncertainty. As we consider MCC to be the most important component of our fitness function, it has a larger weight in Equation 3.

### 4.2. Other Metrics Used for Evaluation

Specificity, a commonly used evaluation metric, can produce misleading scores in datasets with a large number of negative examples (the typical scenario in anomaly detection tasks). Instead of specificity, we have used positive predictive value (also known as precision, Equation (5)). For the sake of brevity, we identify this metric as precision throughout this paper. We also made use of sensitivity (also known as recall, Equation (6)), which we identify as recall throughout this paper. We did not use precision or recall for the optimisation of the hyper-parameters of the detector. They were only used to evaluate the final performance of each method.

(5) $$P=\frac{tp}{tp+fp}$$

(6) $$R=\frac{tp}{tp+fn}$$

### 4.3. Methods Included for Comparison

All methods used were tested on identical test sets $O_{t}$. Once all methods for comparison were carried out, a Wilcoxon test was conducted to compare the precision, recall, and MCC between the three comparison methods and the GRU-AE. These results were corrected for multiple observations using Holm’s method [38].

#### 4.3.1. Luminol

In order to provide a baseline for comparison of performance, LinkedIn’s open source, univariate time-series anomaly detection package, Luminol [39], was used. This library represents out-of-the-box anomaly detection, as well as methods which require data be univariate, often achieved using principal component analysis (PCA). This uses a bitmap representation of a univariate time series, calculated using symbolic aggregate approximation (SAX)—a method for the discretisation of time-series data [40]—to detect anomalies [41]. This is implemented using two concatenated windows, a lagged window and a leading window, which slide across the time series. Each window is then converted into a SAX representation and subsequently into bitmaps based on the frequency of each SAX subword. The distance between the two bitmaps is calculated and represents the anomaly score for the leading window. The calculated anomaly scores are then tested using a grid search-optimised threshold-based anomaly detector in the same way that the GRU-AE is assessed. In order to transform the three environmental variables into a univariate time series, the first principal component was taken.

#### 4.3.2. Time-Series Regression with Autoregression Integrated Moving Average (ARIMA)

Autoregression integrated moving average (ARIMA) is a frequently used model for univariate time-series analysis in statistical analysis. The parameters for this model (*p*, the order of autoregressive model; *d*, the degree of differencing; and *q*, the order of the moving average model) were found using grid search on $H_{t}$, optimising for the lowest Akaike information criteria (AIC). This is a metric used for comparing the goodness of fit of statistical models, though it is not a measure of goodness of fit. Once the optimal parameters were found, $H_{v}$ was used to fit the model. This was then used to forecast 30-minute windows, given the previous 180 minutes in $O_{e}$. Windows where the mean square error between the predicted window and ground truth was beyond some threshold (τ) were considered an alert for the window i+α to i+α+β. Values for τ, α, and β were optimised using grid search and, finally, tested on $O_{t}$. Similar to the Luminol method, the first principal component was taken to transform the three environmental variables into a univariate time series.

#### 4.3.3. Time-Series Regression with GRU Network (GRU-R)

To provide context for how the GRU-AE performs with regards to an alternative deep learning methodology, a GRU network was used for regression. This network was trained to predict future values of the environmental sensors. By training this regression network on only healthy data $H_{t}$, it learned to predict environmental variables for conditions which do not result in occurrences of respiratory disease. The model was trained to predict windows of 30 minutes, the same size as the windows used for the GRU-AE. Similar to the ARIMA model, windows where the mean square error was beyond some threshold (τ) were considered an alert for the window i+α to i+α+β. Similar to the configuration laid out in Section 4.1, these parameters were optimised using PSO, which is trained using $O_{e}$, and finally evaluated on $O_{t}$.

## 5. Results

**Figure 5:**
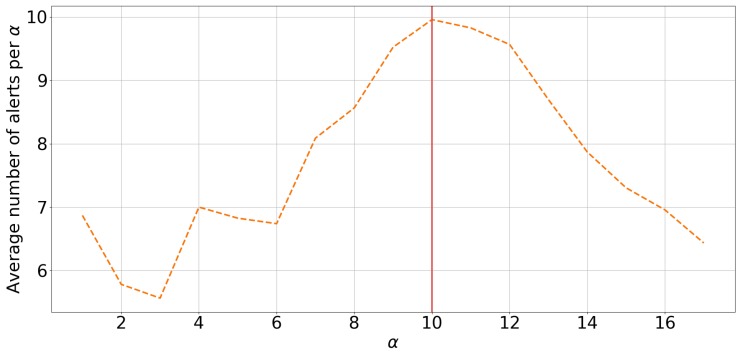
The average number of alerts raised, given an alert window start of α days and a size of β=7, using the optimal threshold τ for each given α found using grid search on the anomaly detection used on Mahalanobis distances produced by a GRU-based autoencoder.

For the purpose of exploration, the GRU-AE’s anomaly detector was initially optimised using a grid search with a fixed β=6 with $O_{v}$ used as the test set. For values from α=10 onwards, there was a steady and sustained decrease in the total number of alerts raised (Figure 5). This was indicative of the point where the acceptable forecast window extended beyond the data available, resulting in an increased portion of alerts that were unverifiable in terms of validity. This decline in alerts signified the maximum range of the acceptable forecast window α+β=16. This maximum endpoint was used to reduce the search space of the PSO, and, therefore, a particle was considered invalid if α+β>16. The search space was also restricted by stipulating that a particle was also invalid if it exceeded the following ranges: 0≤τ≤20, 1≤α≤12, and 1≤β≤6. The limits on τ cover all feasible values; this was found through preliminary testing. β was limited to a maximum of 6, as a range greater than this was deemed to be too large.

As outlined in Section 4.1, grid search was used to find the hyper-parameters for the GRU-AE. The grid search converged to the same set of parameters in almost all batches, so, for the sake of space, we report in Table 1 the most frequently identified values for all hyper-parameters. As expected, the hidden size was very low. This is because under healthy circumstances there is little fluctuation in the environment, and, therefore, only a small amount of memory is required. It is when there are deviations from this—frequent or drastic fluctuations—that occurrences of respiratory disease can be expected.

Table 2 shows the batch-by-batch performance of each of the three methodologies; each row presents the performance of the three compared systems when a given batch is used for testing. The GRU-AE achieves better results than the other methods for all performance metrics measured (precision, recall, and MCC). The most notable difference between the proposed GRU-AE and other methodologies is the substantial shift in performance in terms of precision and recall; ARIMA has also achieved this, but to a much lower degree. In the Luminol and GRU-R models, a higher precision is achieved in comparison to recall, indicating that these models are more conservative in their alerts. However, in the GRU-AE model, the solutions found tend to rely on maximising recall at the expense of precision, $\mu_{P}=0.776,\mu_{R}=0.938$, whilst not sacrificing it so much to be degrading to the model’s overall performance. The substantially higher recall for the GRU-AE model indicates the models capacity to identify negative changes in the environment that lead to an increased number of pigs with symptoms of respiratory disease; the lower precision of the model could indicate that not all negative changes in the environment result in occurrences of respiratory disease. This is backed up by the fact that there are other factors [42] that contribute to respiratory disease prevalence, not just the environmental measures used in this analysis.

The last row of Table 2 contains a high-level quantitative summary of the performance of all methods across batches. We have counted the number of batches in which each method obtains either an incorrect score of 0 for MCC (I), correct score of 1 for MCC (C), or in between 0 and 1 (B). In terms of the C count, Luminol clearly performs the worst of the methods, while the others have a similar score (10–11). At the same time, GRU-AE stands out as having the lowest count of I, twice as low as the score of ARIMA and GRU-R, and almost four times lower than Luminol’s, whilst simultaneously keeping the lowest count of B.

Numerically, at least, we can see that the difference in performance between Luminol and GRU-AE may be considered substantial. However, the results from the Wilcoxon test showed that the difference was not statistically significant for precision (p=0.184), recall (p=0.059), and MCC (p=0.054). The Wilcoxon test also concluded there was no significant difference between the ARIMA model and GRU-AE, and GRU-R and GRU-AE models, in terms of any of the performance metrics.

Looking only at test batches where there were no events (Table 2), the deep learning models excel in determining that there are no alerts to be raised, achieving extremely high precision and recall. This indicates that the GRU is capable of modelling the underlying representation of “normality” very well, as alerts are not raised when they should not be. For batches where the number of events was greater than zero, the ARIMA model outperformed both of the GRU-based models in terms of precision. However, this came at a substantial sacrifice in terms of recall, indicating that the model was likely underpowered. Both of the GRU-based models decreased in performance in terms of precision, but only the GRU-AE was able to retain its high recall. The optimised α and β values for each batch are reported in Table 3.

### 5.1. Case Study

**Figure 6:**
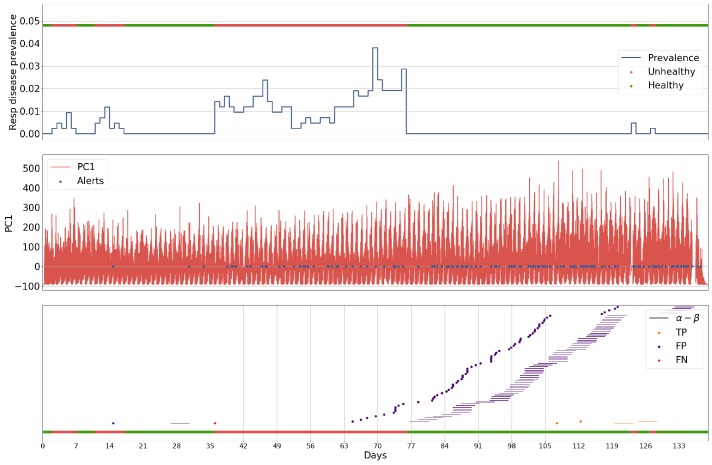
Results for batch 27 processed by the Luminol-powered anomaly detection, given its grid search-optimised parameters α (the start of the window for which an alert is for) and β (the length of the window), in relation to the prevalence of respiratory disease within a single batch.

**Figure 7:**
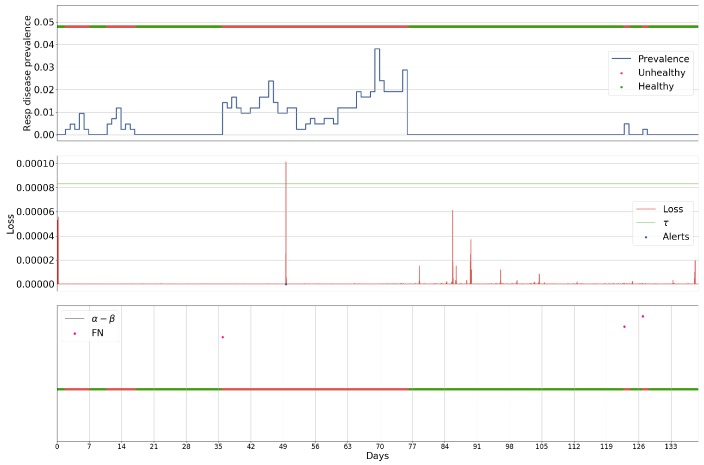
Results for batch 27 processed by the ARIMA-powered anomaly detection, given its grid search-optimised parameters α (the start of the window for which an alert is for), β (the length of the window), and τ (the threshold which the loss between predicted values and actual values must cross to be considered an alert), in relation to the prevalence of respiratory disease within a single batch.

**Figure 8:**
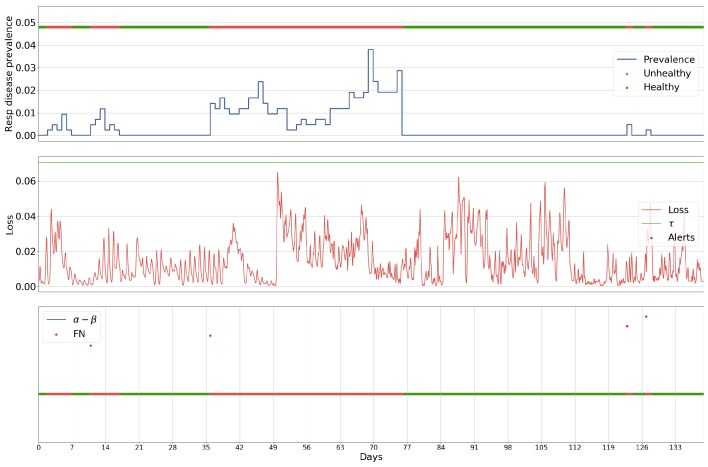
Results for batch 27 processed by the GRU-R-powered anomaly detection, given its PSO-optimised parameters α (the start of the window for which an alert is for), β (the length of the window), and τ (the threshold which the loss between predicted values and actual values must cross to be considered an alert), in relation to the prevalence of respiratory disease within a single batch.

**Figure 9:**
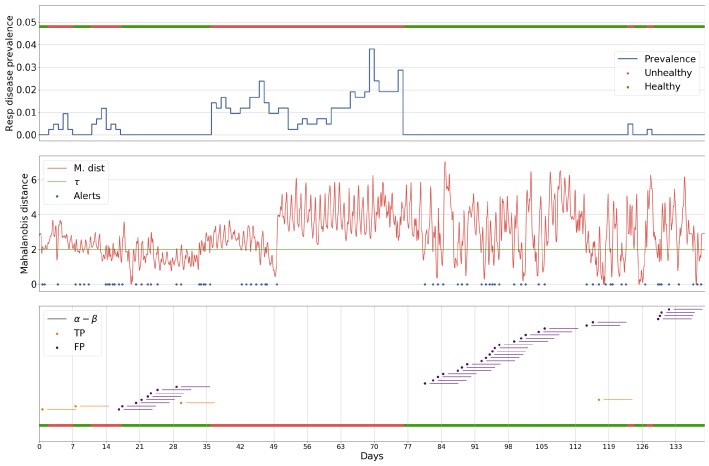
Results for batch 27 processed by the GRU-autoencoder-powered anomaly detection, given its PSO-optimised parameters α (the start of the window for which an alert is for), β (the length of the window), and τ (the threshold which the loss between predicted values and actual values must cross to be considered an alert), in relation to the prevalence of respiratory disease within a single batch.

The following case is presented for batch 27 exemplifies the characteristics of each of the methodologies applied to the problem. This batch was chosen as it contained the highest number of anomalous events in a single batch, making it one of the more challenging batches for a method to perform well on. The batch was analysed by a Luminol-powered, grid search-optimised anomaly detection (Figure 6), an ARIMA-powered, grid search-optimised anomaly detection (Figure 7, a GRU-R-powered, PSO-optimised anomaly detection (Figure 8), and a GRU-AE-powered, PSO-optimised anomaly detection (Figure 9). The first subplot for each figure shows respiratory disease prevalence in both analogue and binary form; the second plots the univariate time series that was used for anomaly detection and the alerts raised for it. In the case of the Luminol model, this was the first principal component of the temperature, humidity, and CO${}_{2}$ time series. For the GRU-R and ARIMA models, the mean square error between predicted and ground truth was used; for the GRU-autoencoder, the binary cross-entropy loss between each input window and the model’s recreation of it was used. The final subplot shows the results of the anomaly detection for each of the alerts, along with their alert windows.

The two deep learning-based models show signs of consistency in the underlying technology, as they both capture similar events within the environmental data, demonstrated by the common peaks and troughs seen in the loss. The Luminol-based model correctly produces alerts to some of the occurrences of respiratory disease prevalence, but these are severely outweighed by the number of false positives that it also produces. The GRU-R and ARIMA models suffer from the inverse problem in that no alerts are raised, even though the latter appears at first glance to produce some significant anomalous points. These points rarely align with actual events, and the success of the ARIMA model is largely down to not raising many alerts at all. The GRU-autoencoder model, however, strikes a balance between these solutions, alerting to almost all of the occurrences of respiratory disease, albeit with some false positives, though far fewer than the Luminol model.

### 5.2. Influence of the Number of Hidden Layers on GRU-AE Performance

**Figure 10:**
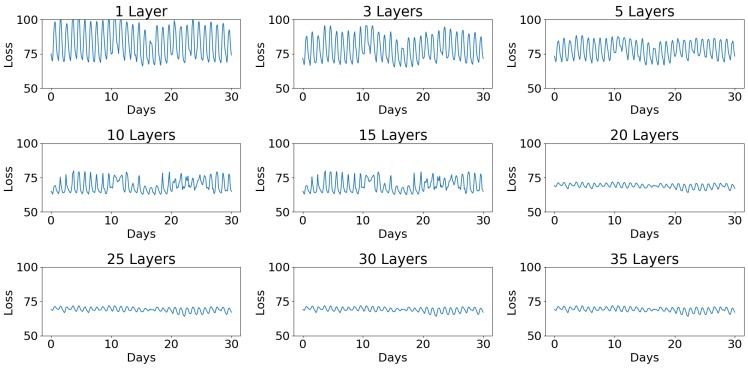
How different numbers of layers in a GRU-AE affect the ability to model environmental data. The smaller the standard deviation, the better the model performs.

The number of layers used in the encoder and decoder had an effect on what the GRU-AE was capable of modelling (Figure 10). Since the concept behind the model was that loss is reduced for “healthy” data, the perfect environment’s loss would have a constant loss over time, therefore meaning the model architecture should be chosen to minimise the standard deviation of the loss in healthy data. Using a small number of layers, 1–15, there was little change in the ability of the GRU-AE to model “healthy data”. Once the number of layers reached 20 for each half of the GRU-AE, the performance reached its maximum and would not reduce the standard deviation of loss any further.

### 5.3. Computation Time

Despite its lacking in performance, the Luminol-based model presented (Figure 6) was trained and optimised in a fraction of the time required to train the two GRU models presented. The Luminol model was fully trained within a period of 6 h and the ARIMA-based model was fully trained within 24 h. These models were run on a machine containing two Xeon E5-2699v4 processors (totalling 44 cores), whereas the two GRU models took a week on a similarly performing machine equipped with an Nvidia GTX 980 Ti.

## 6. Discussion

This paper set out to develop and evaluate a methodology utilising recurrent neural networks comprising GRUs, and to apply the methodology to the early detection of respiratory disease in growing pigs. This network structure was used to build an autoencoder capable of accounting for the temporal aspect of data within the structure of the network itself, rather than solely through the structuring of the data. The GRU-AE specifically outperforms the GRU-R where there were events to be detected, resulting in a substantially higher recall for the GRU-AE.

The novelty of this work lies in the complete reassessment of the idea of a recurrent autoencoder for multi-sensor anomaly detection of Malhotra et al. [3]. The primary adjustments made were switching from LSTM cells, currently the most common unit in recurrent networks, to GRUs. This benefits the network as a whole, as GRUs are less complex and therefore more efficient whilst providing comparable performance [43,44,45,46]. A PSO-optimised anomaly detector was also created for processing the output of the GRU-AE to determine if a window was anomalous or not. In addition to the methodological changes, this work provides a much more in-depth evaluation of the performance of the GRU-AE through the use of a case study and concrete comparisons with alternative methods, whilst also applying the methodology to real-world data.

Given the recent trends in livestock keeping, there is a move towards automating significant portions of livestock management and monitoring in response to the ever-increasing demand for high-quality, sustainable, and safe meat production [47]. These changes in livestock management offer opportunities for automation in disease detection, such as the one developed here. A common approach to embracing what is generally referred to as precision livestock farming (PLF) is the introduction of a variety of sensors. Small, relatively cheap, and high-resolution sensors can monitor environmental variables, such as those assessed in this study, and also capture large quantities of other data using sensors such as cameras [48] and microphones [49]. In the case of disease detection, any false negatives have the potential to lead to epidemics and therefore increase the disease risk of livestock systems. For this reason, a high precision ought to be sacrificed if a higher recall can be achieved, as false positives pose less danger than false negatives in this application. The GRU-AE presented in this paper acts upon this, ensuring that recall is maximised as much as possible.

The motivation for developing such a methodology was to deal with the very low disease prevalence in the data that was collected. In this work, we concentrated on the detection of respiratory diseases. There were several reasons for this, including the fact that the prevalence of respiratory diseases was highest amongst this class of pigs in this dataset. We have similar data for other disorders that affect pigs, such as lameness and digestive diseases, but as their incidence is much lower than respiratory diseases, developing similar methodologies would result in an insufficient number of occurrences to validate the model.

Steps were taken to mitigate the challenges posed by the data, such as summing all classifications of respiratory disease (light, mild, severe, and irrecoverable), as described in Section 3.2. This was justified, as this methodology is for an early-warning system, which only needs to know if there is disease or if there is not. Therefore, looking at respiratory disease prevalence as a whole, rather than segmented into levels of severity, makes for a simpler dataset that is more focussed on the challenge being tackled. It should be noted that the methodology developed here can be applied to all types of respiratory diseases in pigs.

The detection of respiratory disease following a change in the environmental condition fits within the parameters derived from literature, such that we are not predicting oncoming disease before pathogen incubation (e.g., PRRS has an average incubation time of 14 days [32]). Both the GRU-R and GRU-AE are able to raise an alert for oncoming respiratory disease 1–7 days prior to pigs in a batch first showing symptoms. The Luminol model does not reflect this, however, this is likely due to its inability to find hyper-parameters that result in a strong MCC. This causes the PSO to maximise the values for α and β, as there is no repercussion for doing so if the performance is poor regardless.

Due to the renewed popularity of RNN-based models for sequence analysis, a result of their exceptional performance in fields such as natural language processing, there is a need for methods to increase the interpretability of these black-box models. Work in this area has steadily grown [50,51], and future work might consider applying such methods to the model to gain a better understanding of the risk factors for respiratory disease in growing pigs based on the GRU-AE. As this method is assumption-free and works without considering differences between countries, additional work to validate that this multi-sensor anomaly detection scales beyond the domain of health within agriculture, thus validating its robustness, would be valuable to the wider community.

## 7. Conclusions

We have presented a deep learning-based model capable of raising an early warning to occurrences of respiratory disease in growing pigs. The proposed methodology of a GRU-based autoencoder combined with a PSO-optimised anomaly detector shows strong potential in its ability to detect anomalies that will lead to occurrences of respiratory disease in growing pigs, and it is robust to country-specific environments. This GRU-AE was able to outperform other comparable methodologies in precision, recall, and overall performance in both scenarios where there was a presence or an absence of disease.

## Figures and Tables

**Table 1:** The grid search-optimised hyper-parameters used to train a GRU-AE used on multidimensional time-series data.

Hyper-Parameter	Value
Hidden size	4
No of layers	20
Mini-batch size	1024
Dropout	0.5
Learning rate	$1\times 10^{-6}$
Momentum	0.3

**Table 2:** Table of results for the grid search-optimised implementation of LinkedIn’s anomaly detection library (Luminol), the grid search-optimised autoregression integrated moving average (ARIMA) model, particle swarm optimisation (PSO)-optimised threshold-based anomaly detection of loss incurred from a GRU-based regression, and PSO-optimised threshold-based anomaly detection of Mahalanobis distance produced by a GRU-based autoencoder. The events column lists how many times the respiratory disease prevalence increases from 0 within the batch, P denotes the precision, and R is the recall of the model. These results are from the test data $O_{t}$. The final I/B/C row indicates the number of folds that were Incorrect (MCC=0.0), Between (0.0<MCC<1.0), or Correct (MCC=1.0)

ID	Events	Luminol			ARIMA			GRU-R			GRU-AE		
		**P**	**R**	**MCC**	**P**	**R**	**MCC**	**P**	**R**	**MCC**	**P**	**R**	**MCC**
5	0	0.111	0.125	0.118	1.0	1.0	1.0	1.0	1.0	1.0	0.0	0.0	0.0
9	0	1.0	0.1	0.316	1.0	1.0	1.0	1.0	1.0	1.0	1.0	1.0	1.0
10	0	0.0	0.0	0.0	1.0	1.0	1.0	0.0	0.0	0.0	1.0	1.0	1.0
14	0	1.0	1.0	1.0	1.0	1.0	1.0	1.0	1.0	1.0	1.0	1.0	1.0
16	0	1.0	1.0	1.0	0.0	1.0	0.0	1.0	1.0	1.0	1.0	1.0	1.0
17	0	1.0	0.667	0.816	1.0	1.0	1.0	1.0	1.0	1.0	1.0	1.0	1.0
18	0	1.0	1.0	1.0	0.0	1.0	0.0	1.0	1.0	1.0	1.0	1.0	1.0
19	0	1.0	0.667	0.816	1.0	1.0	1.0	1.0	1.0	1.0	1.0	1.0	1.0
24	0	1.0	1.0	1.0	1.0	1.0	1.0	1.0	1.0	1.0	1.0	1.0	1.0
26	4	1.0	0.667	0.816	0.053	0.5	0.162	1.0	0.0	0.0	0.250	1.0	0.500
27	5	0.0	0.0	0.0	1.0	0.0	0.0	0.0	0.0	0.0	0.111	1.0	0.333
28	3	0.0	0.0	0.0	1.0	0.0	0.0	0.0	0.0	0.0	1.0	1.0	1.0
29	1	0.0	0.0	0.0	1.0	1.0	1.0	0.0	0.0	0.0	1.0	1.0	0.0
30	0	0.0	0.0	0.0	0.0	1.0	0.0	1.0	1.0	1.0	1.0	1.0	1.0
31	1	0.0	0.0	0.0	1.0	1.0	1.0	1.0	1.0	1.0	0.050	1.0	0.224
32	0	0.0	0.0	0.0	1.0	1.0	1.0	1.0	1.0	1.0	1.0	1.0	1.0
Avg. Events = 0		0.647	0.505	0.551	0.727	1.000	0.727	0.909	0.909	0.909	0.909	0.909	0.909
Avg. Events > 0		0.200	0.133	0.163	0.811	0.500	0.432	0.400	0.400	0.200	0.482	1.000	0.411
Avg. All		0.507	0.390	0.430	0.753	0.844	0.635	0.750	0.688	0.688	0.776	0.938	0.754
I/B/C				7/5/4			5/1/10			5/0/11			2/3/11

**Table 3:** Table of results for the grid search-optimised implementation of LinkedIn’s anomaly detection library (Luminol), the grid search-optimised ARIMA model, PSO-optimised threshold-based anomaly detection of loss incurred from a GRU-based regression, and PSO-optimised threshold-based anomaly detection of the Mahalanobis distance produced by a GRU-based autoencoder. α and β denote the start and length of the time window (in days), respectively, for which an alert is assessed, and τ is the threshold the loss/Mahalanobis distance needed to exceed in order to raise an alert. They are optimised using training data $O_{e}$; these results are from the test data $O_{t}$.

ID	Events	Luminol		ARIMA		GRU-R		GRU-AE	
		α	β	α	β	α	β	α	β
5	0	12	4	12	1	1	6	1	1
9	0	12	4	12	1	1	6	1	2
10	0	12	4	12	1	1	6	1	2
14	0	12	4	12	1	1	6	1	4
16	0	12	4	12	1	1	6	3	1
17	0	12	4	12	1	1	6	1	5
18	0	12	4	12	1	1	6	3	5
19	0	12	4	12	1	1	6	1	5
24	0	12	4	12	1	1	6	1	6
26	4	12	4	12	1	2	6	3	6
27	5	12	4	12	4	4	6	1	6
28	3	12	4	12	1	5	6	8	6
29	1	12	4	12	2	1	6	2	6
30	0	12	4	12	1	1	6	4	6
31	1	12	4	12	1	1	6	1	6
32	0	12	4	12	1	1	6	8	5

## References

[1] Huhn R. (1970). Swine enzootic pneumonia: Incidence and effect on rate of body weight gain. Am. J. Vet. Res..

[2] Stärk K.D. (2000). Epidemiological investigation of the influence of environmental risk factors on respiratory diseases in swine—A literature review. Vet. J..

[3] Malhotra P., Ramakrishnan A., Anand G., Vig L., Agarwal P., Shroff G. LSTM-based Encoder-Decoder for Multi-sensor Anomaly Detection. https://arxiv.org/abs/1607.00148.

[4] Malhotra P., Vig L., Shroff G., Agarwal P. (2015). Long short term memory networks for anomaly detection in time series. Proceedings.

[5] Morales I.R., Cebrián D.R., Blanco E.F., Sierra A.P. (2016). Early warning in egg production curves from commercial hens: A SVM approach. Comput. Electron. Agric..

[6] Singh R.K., Sharma V.C. (2015). Ensemble Approach for Zoonotic Disease Prediction Using Machine Learning Techniques.

[7] Valdes-Donoso P., VanderWaal K., Jarvis L.S., Wayne S.R., Perez A.M. (2017). Using Machine learning to Predict swine Movements within a regional Program to improve control of infectious Diseases in the US. Front. Vet. Sci..

[8] Cabral G.G., de Oliveira A.L.I. One-class Classification for heart disease diagnosis. Proceedings of the IEEE International Conference on Systems, Man and Cybernetics (SMC).

[9] Leng Q., Qi H., Miao J., Zhu W., Su G. (2015). One-class classification with extreme learning machine. Math. Probl. Eng..

[10] Thompson R., Matheson S.M., Plötz T., Edwards S.A., Kyriazakis I. (2016). Porcine lie detectors: Automatic quantification of posture state and transitions in sows using inertial sensors. Comput. Electron. Agric..

[11] Münz G., Li S., Carle G. (2017). Traffic anomaly detection using k-means clustering. GI/ITG Workshop MMBnet.

[12] Ambusaidi M.A., Tan Z., He X., Nanda P., Lu L.F., Jamdagni A. (2014). Intrusion detection method based on nonlinear correlation measure. Int. J. Internet Protoc. Technol..

[13] Aghabozorgi S., Shirkhorshidi A.S., Wah T.Y. (2015). Time-series clustering—A decade review. Inf. Syst..

[14] Wong W.K., Moore A.W., Cooper G.F., Wagner M.M. Bayesian network anomaly pattern detection for disease outbreaks. Proceedings of the 20th International Conference on Machine Learning (ICML-03).

[15] Wong W.K., Moore A., Cooper G., Wagner M. (2005). What’s strange about recent events (WSARE): An algorithm for the early detection of disease outbreaks. J. Mach. Learn. Res..

[16] Chauhan S., Vig L. Anomaly detection in ECG time signals via deep long short-term memory networks. Proceedings of the IEEE International Conference on Data Science and Advanced Analytics (DSAA).

[17] Haque S.A., Rahman M., Aziz S.M. (2015). Sensor anomaly detection in wireless sensor networks for healthcare. Sensors.

[18] Veeravalli B., Deepu C.J., Ngo D. (2017). Real-Time, Personalized Anomaly Detection in Streaming Data for Wearable Healthcare Devices. Handbook of Large-Scale Distributed Computing in Smart Healthcare.

[19] Kanarachos S., Mathew J., Chroneos A., Fitzpatrick M. Anomaly detection in time series data using a combination of wavelets, neural networks and Hilbert transform. Proceedings of the 6th IEEE International Conference on Information, Intelligence, Systems and Applications (IISA2015).

[20] Li J., Pedrycz W., Jamal I. (2017). Multivariate time series anomaly detection: A framework of Hidden Markov Models. Appl. Soft Comput..

[21] Chuah M.C., Fu F. ECG anomaly detection via time series analysis. Proceedings of the International Symposium on Parallel and Distributed Processing and Applications.

[22] Chen X.W., Lin X. (2014). Big data deep learning: Challenges and perspectives. IEEE Access.

[23] Kamilaris A., Prenafeta-Boldú F.X. (2018). Deep learning in agriculture: A survey. Comput. Electron. Agric..

[24] Veen F.V. (2016). A Mostly complete chart of Neural Networks.

[25] Lv Y., Duan Y., Kang W., Li Z., Wang F.Y. (2015). Traffic flow prediction with big data: A deep learning approach. IEEE Trans. Intell. Transp. Syst..

[26] Andrews J.T., Morton E.J., Griffin L.D. (2016). Detecting anomalous data using auto-encoders. Int. J. Mach. Learn. Comput..

[27] Rahman A., Smith D., Hills J., Bishop-Hurley G., Henry D., Rawnsley R. A comparison of autoencoder and statistical features for cattle behaviour classification. Proceedings of the International Joint Conference on Neural Networks (IJCNN).

[28] Al Moubayed N., Breckon T., Matthews P., McGough A.S. Sms spam filtering using probabilistic topic modelling and stacked denoising autoencoder. Proceedings of the International Conference on Artificial Neural Networks.

[29] Zoetis (2012). Individual Pig Care.

[30] Alert G. Application Areas. http://www.general-alert.com/Applications.

[31] Whittemore C.T., Kyriazakis I. (2006). Whittemore’s Science and Practice of Pig Production.

[32] The World Organisation for Animal Health (2014). Infection With Porcine Reproductive and Respiratory Syndrome Virus.

[33] Ioffe S., Szegedy C. (2015). Batch normalization: Accelerating deep network training by reducing internal covariate shift. arXiv.

[34] Sutskever I., Vinyals O., Le Q.V. (2014). Sequence to sequence learning with neural networks. Advances in Neural Information Processing Systems.

[35] Tieleman T., Hinton G. (2012). Lecture 6.5-rmsprop: Divide the gradient by a running average of its recent magnitude. COURSERA Neural Netw. Mach. Learn..

[36] Cleveland W.S. (1979). Robust locally weighted regression and smoothing scatterplots. J. Am. Stat. Assoc..

[37] Shi Y., Eberhart R. A modified particle swarm optimizer. Proceedings of the IEEE International Conference on Evolutionary Computation Proceedings, IEEE World Congress on Computational Intelligence.

[38] Holm S. (1979). A simple sequentially rejective multiple test procedure. Scand. J. Stat..

[39] LinkedIn (2016). Luminol. https://github.com/linkedin/luminol.

[40] Lin J., Keogh E., Wei L., Lonardi S. (2007). Experiencing SAX: A novel symbolic representation of time series. Data Min. Knowl. Discov..

[41] Wei L., Kumar N., Lolla V.N., Keogh E.J., Lonardi S., Ratanamahatana C.A. Assumption-Free Anomaly Detection in Time Series. Proceedings of the International Conference on Scientific and Statistical Database Management.

[42] Mortensen S., Stryhn H., Søgaard R., Boklund A., Stärk K.D., Christensen J., Willeberg P. (2002). Risk factors for infection of sow herds with porcine reproductive and respiratory syndrome (PRRS) virus. Prev. Vet. Med..

[43] Chung J., Gulcehre C., Cho K., Bengio Y. Empirical evaluation of gated recurrent neural networks on sequence modeling. https://arxiv.org/abs/1412.3555.

[44] Chung J., Gulcehre C., Cho K., Bengio Y. Gated feedback recurrent neural networks. Proceedings of the International Conference on Machine Learning.

[45] Karpathy A., Johnson J., Li F.-F. Visualizing and understanding recurrent networks. https://arxiv.org/abs/1506.02078.

[46] Jozefowicz R., Zaremba W., Sutskever I. An empirical exploration of recurrent network architectures. Proceedings of the International Conference on Machine Learning.

[47] Berckmans D. (2014). Precision livestock farming technologies for welfare management in intensive livestock systems. Sci. Tech. Re. Off. Int. Epizoot..

[48] Matthews S.G., Miller A.L., Clapp J., Plötz T., Kyriazakis I. (2016). Early detection of health and welfare compromises through automated detection of behavioural changes in pigs. Vet. J..

[49] Chung Y., Oh S., Lee J., Park D., Chang H.H., Kim S. (2013). Automatic detection and recognition of pig wasting diseases using sound data in audio surveillance systems. Sensors.

[50] Krakovna V., Doshi-Velez F. Increasing the interpretability of recurrent neural networks using hidden Markov models. https://arxiv.org/abs/1606.05320v2.

[51] Samek W., Wiegand T., Müller K.R. Explainable Artificial Intelligence: Understanding, Visualizing and Interpreting Deep Learning Models. https://arxiv.org/abs/1708.08296.

